# Treatment of Compound Fractures and Wounds of Joints

**Published:** 1882-03-20

**Authors:** F. C. G. Griffin

**Affiliations:** Surgeon to the Weymouth Royal Hospital; England


### TREATMENT OF COMPOUND FRACTURES AND WOUNDS OF JOINTS BY GLYCERINE AND CARBOLIC ACID.

BY F. C. G. GRIFFIN, A. M., M. B. OXON., M. R. C. S., ENG.,
Surgeon to the Weymouth Royal Hospital.

As various modes of antiseptic treatment continue to be brought forward, and the most opposite opinions are still held as to their merits, I have thought an account of a few cases that I have treated with the glycerinum acidi carbolici of the British Pharmacopœia, without antiseptic spray or any very elaborate precautions, would be of general interest. And although the number of cases is not large, still the fact that they have all got well has produced a strong impression on my mind of the value of the mode of treatment.

A pipe manufacturer, of intemperate habits, who had failed in business and taken to fishing, was engaged in hauling in a net, with others, after dark, when he got his leg caught by a loop of rope and was thrown down, and found himself unable to ri e. The fishermen carried him home, but handled him rather roughly, as they thought he was not seriously hurt. When I saw him I found he had sustained a compound fracture of the tibia, at the junction of the lower and middle thirds, and had lost a large quantity of blood from a small wound caused by the upper fragment of the tibia having been driven through the skin. I bound it up temporarily, to arrest the bleeding. After about twelve hours, when the bleeding had at length quite stopped, the temporary dressing was removed, and a pad of lint soaked in collodion applied. This pad remained on for two days, when it became partly detached, and free oozing of bloody fluid commenced from the wound. I now applied a pad of four thicknesses of lint saturated with glycerinum acidi carbolici to the wound, and a few turns of bandage over it, so as to keep it in its proper position. The lint became firmly adherent to the wound, and the next day I applied a larger pad of four thicknesses of lint soaked in the same way over the original pad, so as to keep it still saturated.

On the third day afterward I cut the edges of the two outer layers of the pad next to the wound, and removed and soaked them with glycerinum acidi carbolici, and then re-applied them, and then the bandage as before. I will here state that I regard it as of much importance to the success of this plan of treatment not to disturb the layers of lint immediately covering and generally attached to the wound. After this treatment had been continued about ten days, a large blister containing dark fluid formed under the pads and showed at their edges. I now cautiously tried whether the under pad was still adherent, and finding it was not, I removed it and found that the wound had healed. I left the blister exposed to the air, and it dried up in a few days. The remaining progress of the case in no way differed from that of one of simple fracture, and the man ultimately completely recovered.

I was informed by this man's wife that previous to the accident he lived almost entirely on beer, and took scarcely any solid food except a little bread, and that if she provided him with a good dinner he used to sell or exchange it for more beer. While he was under my treatment I limited him to a pint of stout daily.

The next patient treated in this way was a boy of about ten years of age, with compound fracture of the tibia, the upper fragment of the bone having been driven through the skin. The glycerinum acidi carbolici was applied on four thicknesses of lint about two hours after the accident, and covered with cotton-wool, and fresh glycerine and acid was applied to the lint daily, without disturbing the layers next to the wound. After about ten days the lint was removed, and the wound found to have healed. A speedy recovery followed. The fracture apparatus used was of the same kind as in the following case.

The next patient was a tradesman who had jumped out of a cart while his horse was running away. He, in consequence, received a simple fracture of the fibula, and a compound dislocation of the foot outward, the lower extremity of the tibia being driven through the skin, the sock, and the elastic of his boot against the ground, and the internal malleolus broken off. When he had been conveyed home the bone was still protruding, and the wound could not be got at until his boot and sock had been cut away. The bone, being covered with dirt from the road, was now carefully cleansed, and with the aid of two other surgeons, who had been sent for at the same time as myself, and arrived soon after, an attempt to reduce the dislocation was made. We did not, however, succeed in effecting the reduction until a slice of bone had been sawn off the projecting end of the tibia. After the reduction the limb was placed on an iron back splint, with two wooden slide splints duly padded and suspended from a cradle, the apparatus being of the kind used at St. Bartholomew's Hospital, in the wards of Sir James Paget when he was surgeon there, and supplied by Ferguson, the surgical-instrument maker. About three hours after the accident I syringed out the ankle-joint with a solution of carbolic acid in thirty-nine parts of recently boiled water, and then, after cleaning around the wound, applied a pad of lint of six or eight thicknesses, saturated with glycerinum acidi carbolici, taking care that the upper layers were of sufficient size to project some little way beyond the wound, so as to exclude air effectually, in case of the patient becoming restless. This was then secured by a bandage. The next day all the upper layers of lint were removed, soaked as before, and then re-applied, except the three next the wound, which were left undisturbed. Then over the lint I put a large piece of carbolic acid plaster, and secured it with a bandage. This mode of dressing was repeated night and morning for several weeks, during the whole of which time not more than about a tablespoonful of discharge escaped from beneath the pad of lint. The discharge was of a pink color, opaque, and nearly solid. The bowels were confined, and pain and starting relieved with opium, for about a fortnight. After this the patient, who ate heartily his ordinary diet of meat, etc., the whole time, used to sit up in bed and write letters, and keep the account relating to his business. After six weeks I gradually reduced the amount of the carbolic acid by adding more glycerine, and when the wound was nearly healed I used spermacetic ointment. He ultimately made a good recovery, and can walk considerable distance with the aid of two sticks.

The next patient was a cab-driver, aged about fifty years. His horse fell down as he was driving, and while endeavoring to hold him up, he was pulled off his seat and broke his leg. He was then taken to the Weymouth Royal Hospital, distant seven miles from the scene of the accident. On his arrival there, I found that he had a lacerated wound about three inches long, through which the upper half of the tibia was protruding. After the fracture had been set, and the edges of the wound had been drawn together, except over the seat of the fracture, where, in consequence of the leg, the skin would not meet without more force being used than appeared desirable, a pad of lint about four thicknesses was saturated with carbolic acid and glycerine, and lightly bandaged on. The fracture apparatus used consisted of an iron back-splint, with two wooden side-splints, padded and suspended from a cradle, as in the previous case. Over the pad a piece of carbolic-acid plaster was placed. The next day a fresh pad of about four thicknesses was soaked as before mentioned, and applied over the previous one, and the plaster over them both. This dressing was repeated night and morning for about a fortnight, after which it was reduced to once a day. The man was on ordinary diet throughout, and there was no constitutional disturbance. About a month after the accident the lint next the wound was for the first time removed, and the wound found to have healed, except over the end of the bone, where there was a wound about an inch long, with bare bone exposed. The special treatment was now discontinued, and poultices were used. A little later a thin layer of bone came away, and the wound then soon healed, and then recovered with a useful leg.

The next patient was a brewer's drayman, a large made and very fat man, accustomed to the free use of the beverages he supplied. His horse started off while he was in a public house, and when he ran to their heads and endeavored to stop them he was knocked down, and before they could be stopped the front wheel of the dray going over inner side of his knee, turned back a large flap of skin, and made a larcerated wound that extended into the knee-joint. A surgeon was called, and gave the foregoing account on resigning the charge of the case. When I saw him a large pad of four thicknesses of lint, saturated with the glycerinum acidi carbolici, was applied over the wound, and kept saturated by fresh supplies on its outer surface renewed daily. For a week or ten days all went well, and no trouble connected with the joint occurred afterward, but at the end of that time the lint came off, and poultices were used instead, the edges of the skin flap being found to be sloughing, and erysipelas of the leg commencing. The erysipelas followed a severe course, as it did also in several other cases that occurred about the same time, but in the end he recovered and returned to his occupation as drayman.

The next patient was a builder's workman, who fell from a scaffold eighteen feet high, thereby sustaining a severe compound fracture of the lower jaw, while another man falling upon him broke his thigh, and the bone coming through the skin wounded the internal saphenous vein, and caused such copious bleeding that the man appeared in danger of immediate death from loss of blood. Under these circumstances a large sponge was bound tightly over the wound, and the bleeding thus arrested. The fracture was then set, a long splint and bandages being used in the ordinary way. The treatment having reached this stage when I first saw the man, was cold, perspiring profusely, with livid face, and evidently almost dying from loss of blood, I applied the glycerinum acidi carbolici freely to the bandages over the sponge, and then lightly bound over them four thicknesses of lint saturated with it. The next day I removed the lint, cut slits at short intervals in the bandages, and ejected the glycerine through them with a syringe and along the upper edge of the sponge, and then reapplied the pad of lint freshly satuarted as before. This treatment was continued for a fortnight without disturbing the sponge, after which the sponge was removed, and the wound found to have healed. The man's health improved throughout, and he recovered in about the same time as if it had been a simple fracture of the leg.—*Lancet, June 18, 1881, p. 985.—Braithwait.*

